# Supplementary material for: Two New Alkaloids and a New Butenolide Derivative from the Beibu Gulf Sponge-Derived Fungus Penicillium sp. SCSIO 41413
Source: Mar Drugs. 2022 Dec 29;21(1):27. doi: 10.3390/md21010027 (PMC9864330; doi:10.3390/md21010027)
Supplement: Supplementary file 1 [file marinedrugs-21-00027-s001.zip › marinedrugs-2101051-supplementary.pdf]

SUPPLEMENTARY MATERIAL

Two new alkaloids and a new butenolide derivative from the Beibu Gulf sponge-derived fungus *Penicillium* sp. SCSIO 41413

Yuxiu Ye[1], Jiaqi Liang[2], Jianglian She[2], Xiuping Lin[2], Junfeng Wang[2], Yonghong Liu[1,2], Dehua Yang[3], Yanhui Tan[4,*], Xiaowei Luo[1,*], Xuefeng Zhou[1,2,*]

1 Institute of Marine Drugs, Guangxi University of Chinese Medicine, Nanning 530200, China;
2 CAS Key Laboratory of Tropical Marine Bio-resources and Ecology/Guangdong Key Laboratory of Marine Materia Medica, South China Sea Institute of Oceanology, Chinese Academy of Sciences, Guangzhou 510301, China;
3 The National Center for Drug Screening, Shanghai Institute of Materia Medica, Chinese Academy of Sciences, Shanghai 201203, China;
4 State Key Laboratory for Chemistry and Molecular Engineering of Medicinal Resources, School of Chemistry and Pharmaceutical Sciences, Guangxi Normal University, Guilin 541001, China
* Correspondence: tyh533@126.com (Y.T.); luoxiaowei1991@126.com (X.L.); xfzhou@scsio.ac.cn (X.Z.); Tel.: +86-020-89023174 (X.Z.)

Abstract: Marine sponge-derived fungi have been proven to be a prolific source of bioactive natural products. Two new alkaloids, polonimides E (**1**) and D (**2**), and a new butenolide derivative, eutypoid F (**11**), were isolated from the Beibu Gulf sponge-derived fungus, *Penicillium* sp. SCSIO 41413, together with thirteen known compounds (**3–10, 12–16**). Their structures were determined by detailed NMR, MS spectroscopic analyses, and electronic circular dichroism (ECD) analyses. Butenolide derivatives **11** and **12** exhibited inhibitory effect against the enzyme PI3K with IC_{50} values of 1.7 μM and 9.8 μM, respectively. The molecular docking was also performed to understand the inhibitory activity, while **11** and **12** showed obvious protein/ligand-binding effects to the PI3K protein. Moreover, **4** and **15** displayed obvious inhibitory activity against LPS-induced NF-κB activation in RAW264.7 cells at 10 μM.

Keywords: Sponge-derived fungus; *Penicillium* sp.; PI3K; NF-κB

Contents

The ITS gene sequence data of *Penicillium* sp. SCSIO 41413

Figure S1. The ^{1}H NMR spectrum of compound 1 in DMSO.

Figure S2. The ^{13}C NMR spectrum of compound 1 in DMSO.

Figure S3. The HSQC spectrum of compound 1 in DMSO.

Figure S4. The HMBC spectrum of compound 1 in DMSO.

Figure S5. The ^{1}H-^{1}H COSY spectrum of compound 1 in DMSO.

Figure S6. The HRESIMS spectrum of compound 1 in DMSO.

Figure S7. The IR spectrum of compound 1 in DMSO.

Figure S8. The UV and ECD spectrum of compound 1 in DMSO.

Figure S9. The experimental ECD spectrum of compound 1.

Figure S10.The ^{1}H NMR spectrum of compound 2 in DMSO.

Figure S11. The ^{13}C NMR spectrum of compound 2 in DMSO.

Figure S12. The HSQC spectrum of compound 2 in DMSO.

Figure S13. The HMBC spectrum of compound 2 in DMSO.

Figure S14. The ^{1}H-^{1}H COSY spectrum of compound 2 in DMSO.

Figure S15. The HRESIMS spectrum of compound 2 in DMSO.

Figure S16. The IR spectrum of compound 2 in DMSO.

Figure S17. The UV spectrum of compound 2 in DMSO.

Figure S18. The ^{1}H NMR spectrum of compound 11 in DMSO.

Figure S19. The ^{13}C NMR spectrum of compound 11 in DMSO.

Figure S20. The HSQC spectrum of compound 11 in DMSO.

Figure S21. The HMBC spectrum of compound 11 in DMSO.

Figure S22. The ^{1}H-^{1}H COSY spectrum of compound 11 in DMSO.

Figure S23. The HRESIMS spectrum of compound 11 in DMSO.

Figure S24. The IR spectrum of compound 11 in DMSO.

Figure S25. The UV spectrum of compound 11 in DMSO.

Figure S26. The experimental ECD spectrum of compound 12 in DMSO.

The physicochemical data of the known compounds 3~10 and 12~16

The ITS gene sequence data of *Penicillium* sp. SCSIO 41413

GATATGCTTAAGTTCAGCGGGTATCCCTACCTGATCCGAGGTCAACCTGGATAAAAATT

TGGGTTGATCGGCAAGCGCCGGCCGGGCCTACAGAGCGGGTGACAAAGCCCCATACG

CTCGAGGACCGGACGCGGTGCCGCCGCTGCCTTTCGGGCCCGTCCCCCGGAATCGGA

GGACGGGGCCCAACACACAAGCCGGGCTTGAGGGCAGCAATGACGCTCGGACAGGC

ATGCCCCCCGGAATACCAGGGGGCGCAATGTGCGTTCAAAGACTCGATGATTCACTGA

ATTTGCAATTCACATTACGTATCGCATTTCGCTGCGTTCTTCATCGATGCCGGAACCAAG

AGATCCGTTGTTGAAAGTTTTAAATAATTTATATTTTCACTCAGACTTCAATCTTCAGAC

AGAGTTCGGGGGTGTCTTCGGCGGGCGCGGGCCCGGGGGCGTGAGCCCCCCGGCGGC

CAGTAAAGGCGGGCCCGCCGAAGCAACAAGGTAAAATAAACACGGGTGGGAGGTTGG

ACCCAAAGGGCCCTCACTCGGTAATGATCCTTCCGCA

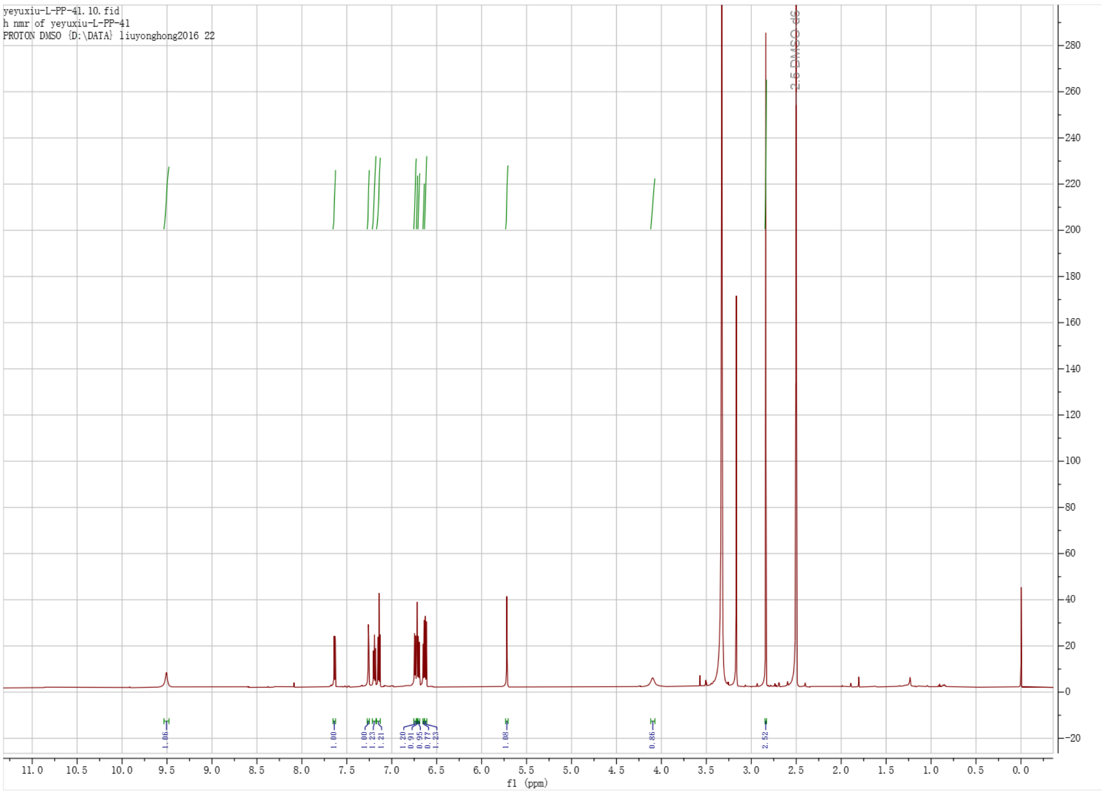

Figure S1. The ^{1}H NMR spectrum of compound 1 in DMSO.

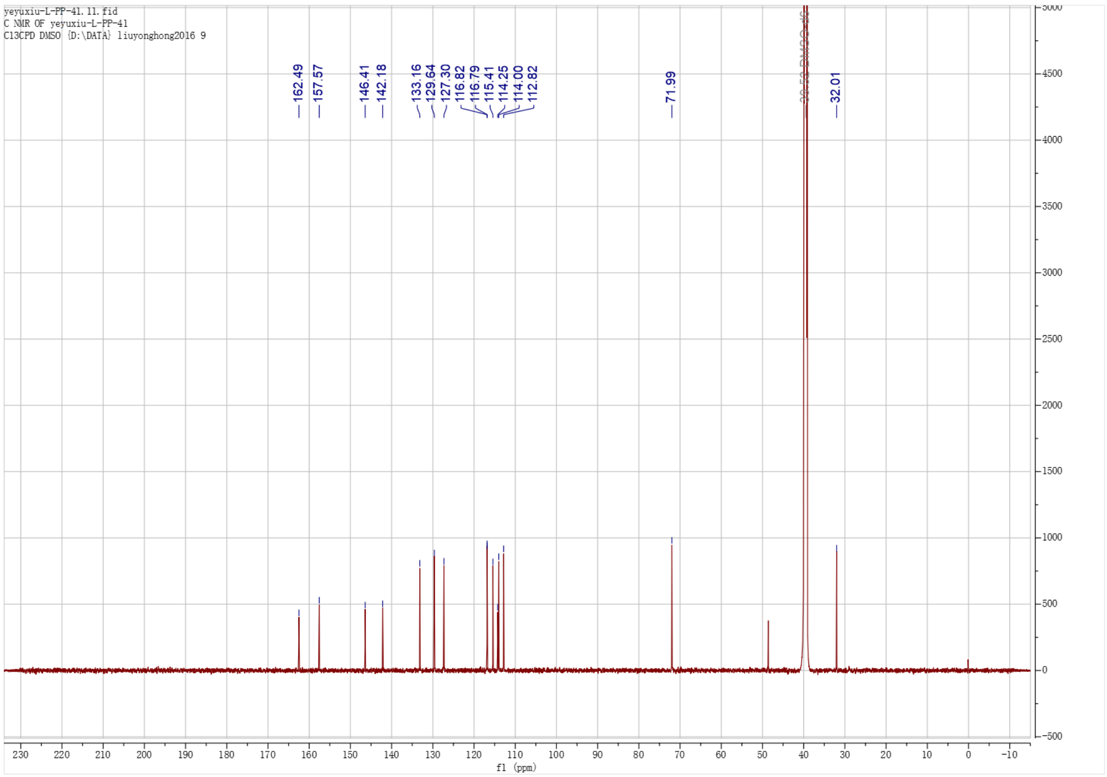

Figure S2. The ^{13}C NMR spectrum of compound 1 in DMSO.

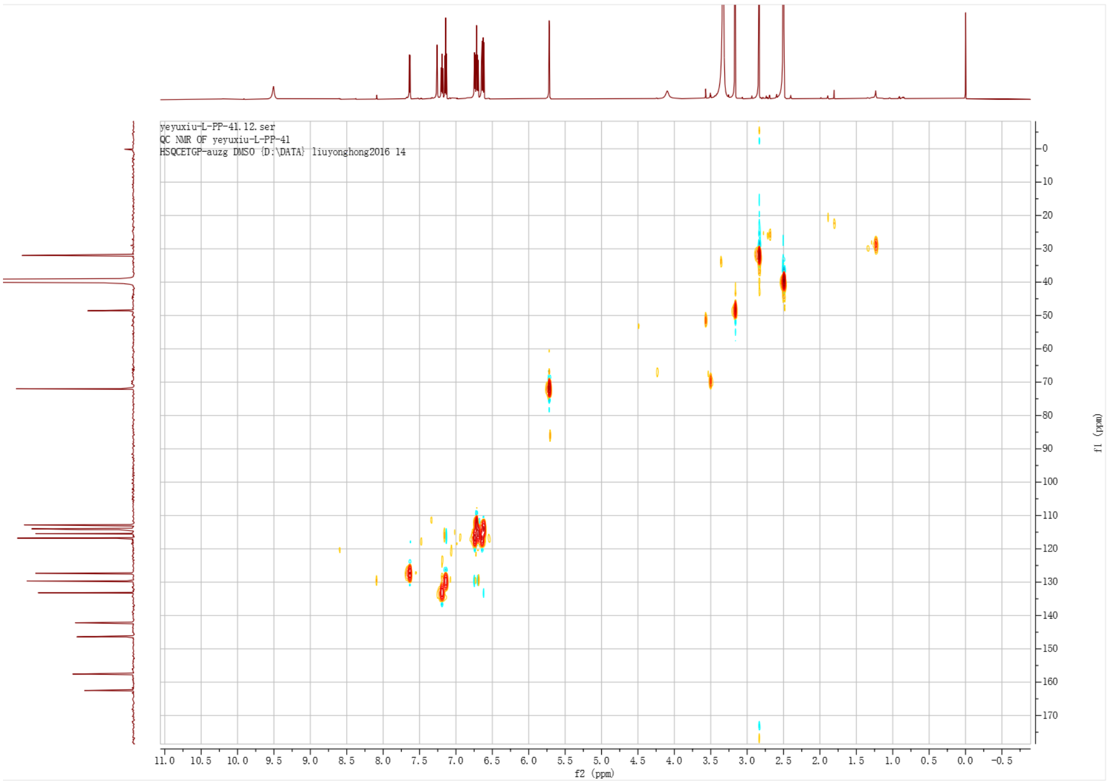

Figure S3. The HSQC spectrum of compound 1 in DMSO.

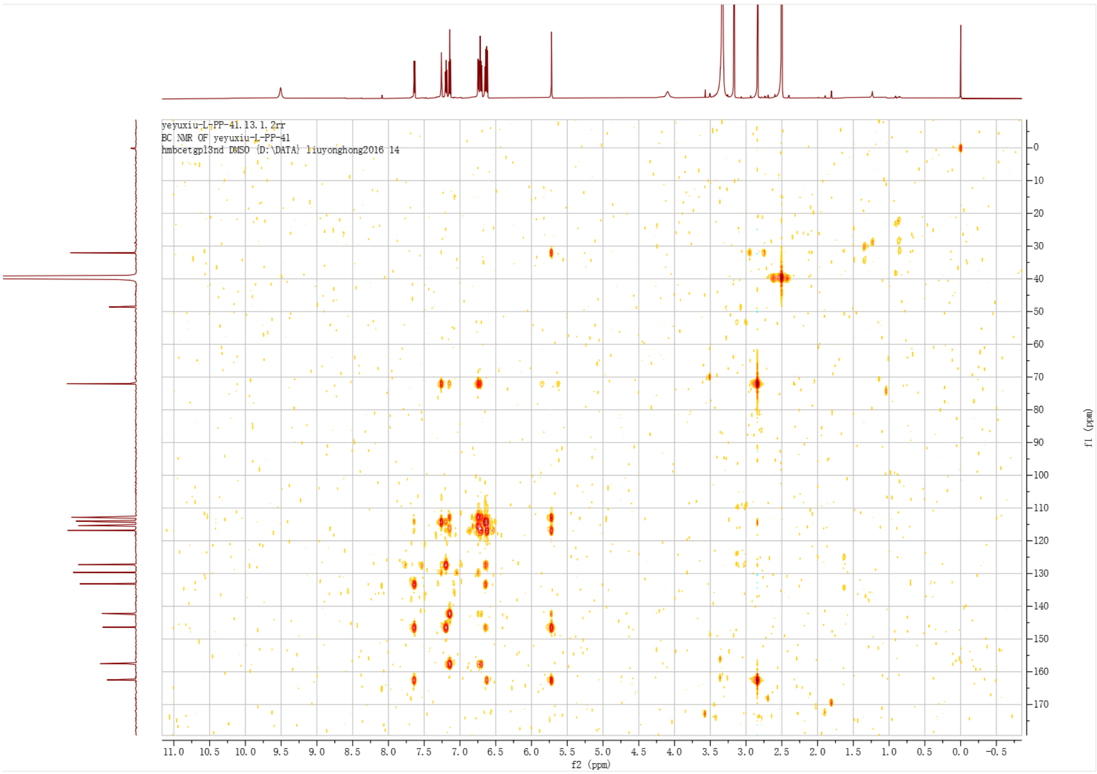

Figure S4. The HMBC spectrum of compound 1 in DMSO.

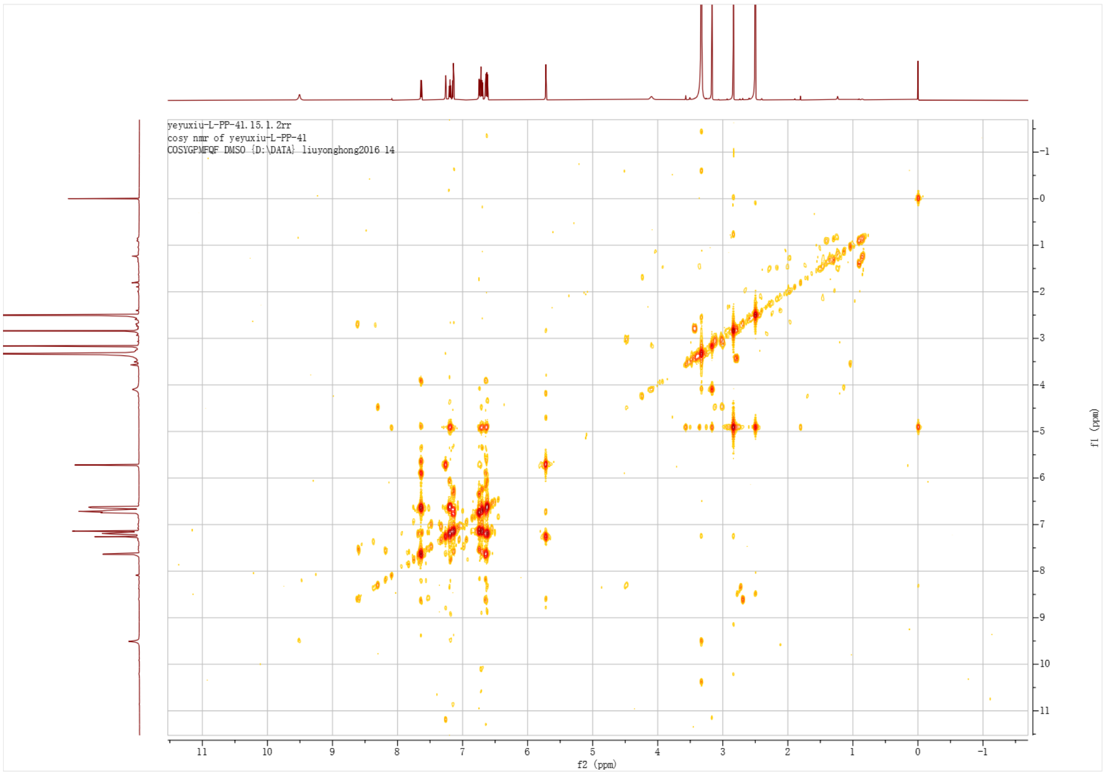

Figure S5. The ^1H-^1H COSY spectrum of compound 1 in DMSO.

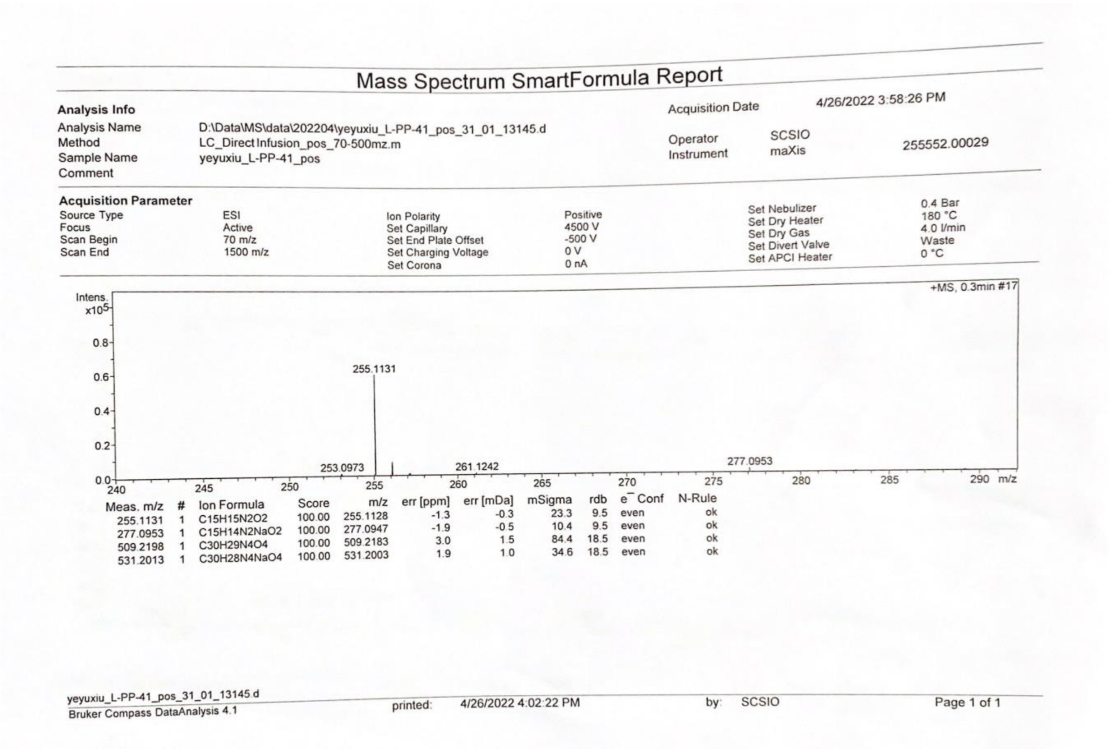

Figure S6. The HRESIMS spectrum of compound 1 in DMSO.

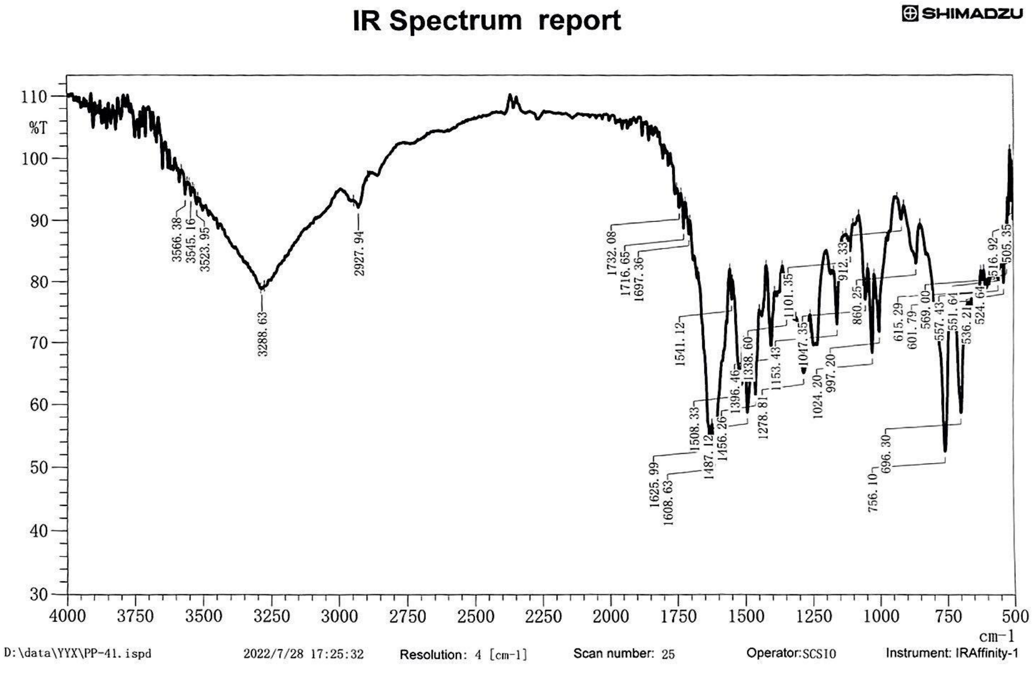

Figure S7. The IR spectrum of compound 1 in DMSO.

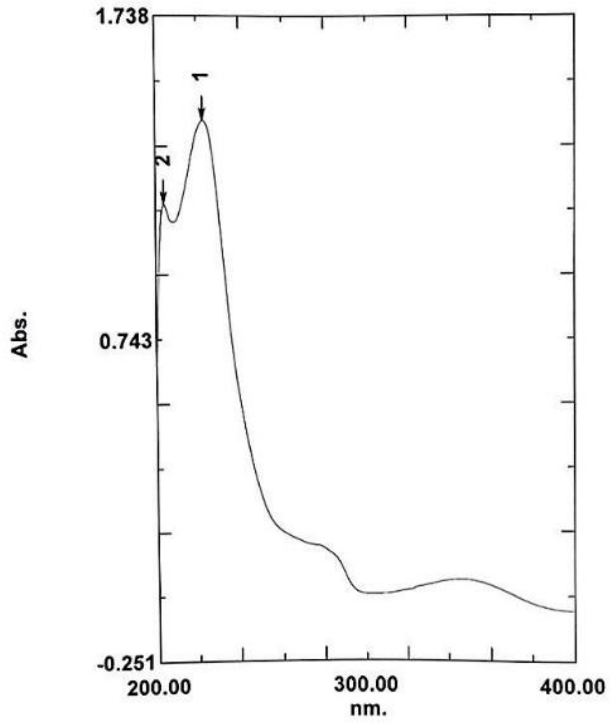

Figure S8. The UV spectrum of compound 1 in DMSO.

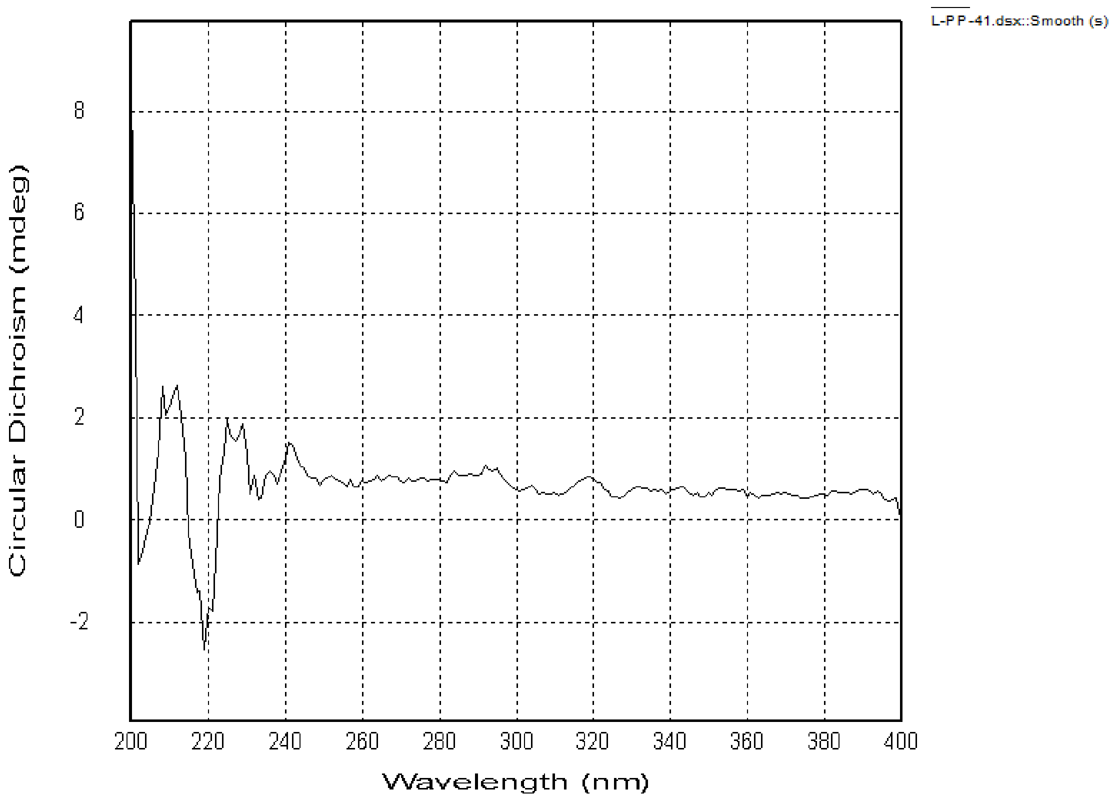

Figure S9. The experimental ECD spectrum of compound 1.

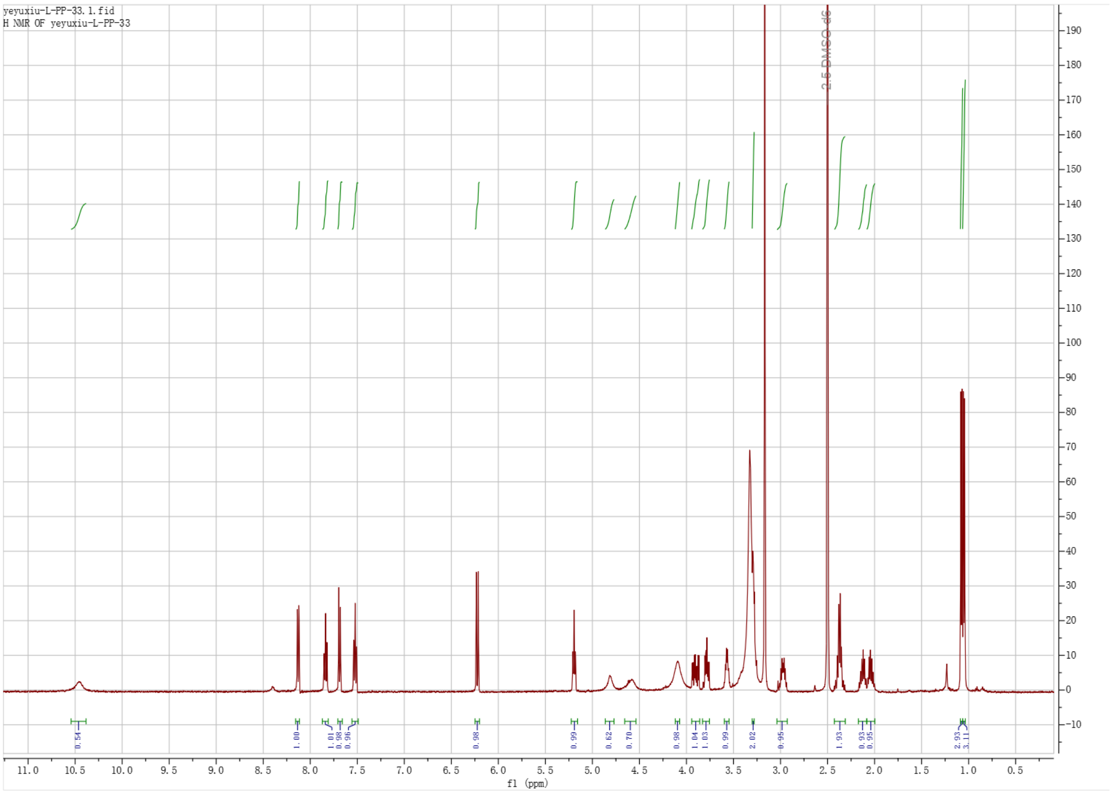

Figure S10. The ^{1}H NMR spectrum of compound 2 in DMSO.

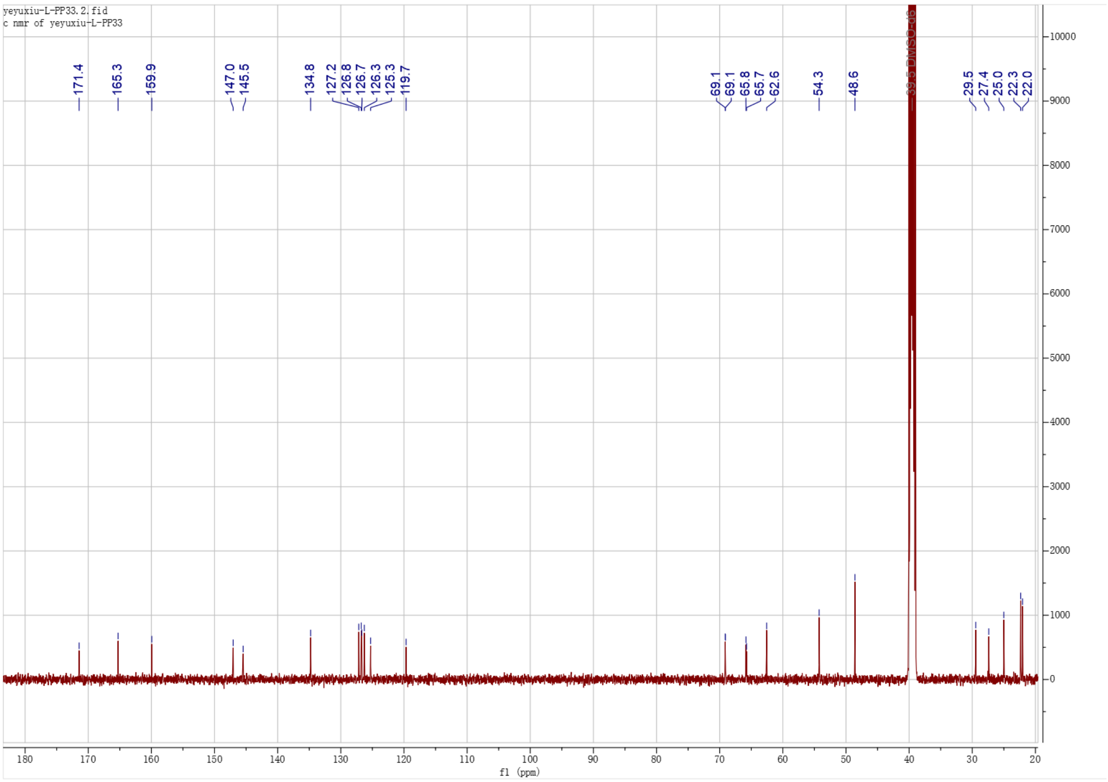

Figure S11. The ^{13}C NMR spectrum of compound 2 in DMSO.

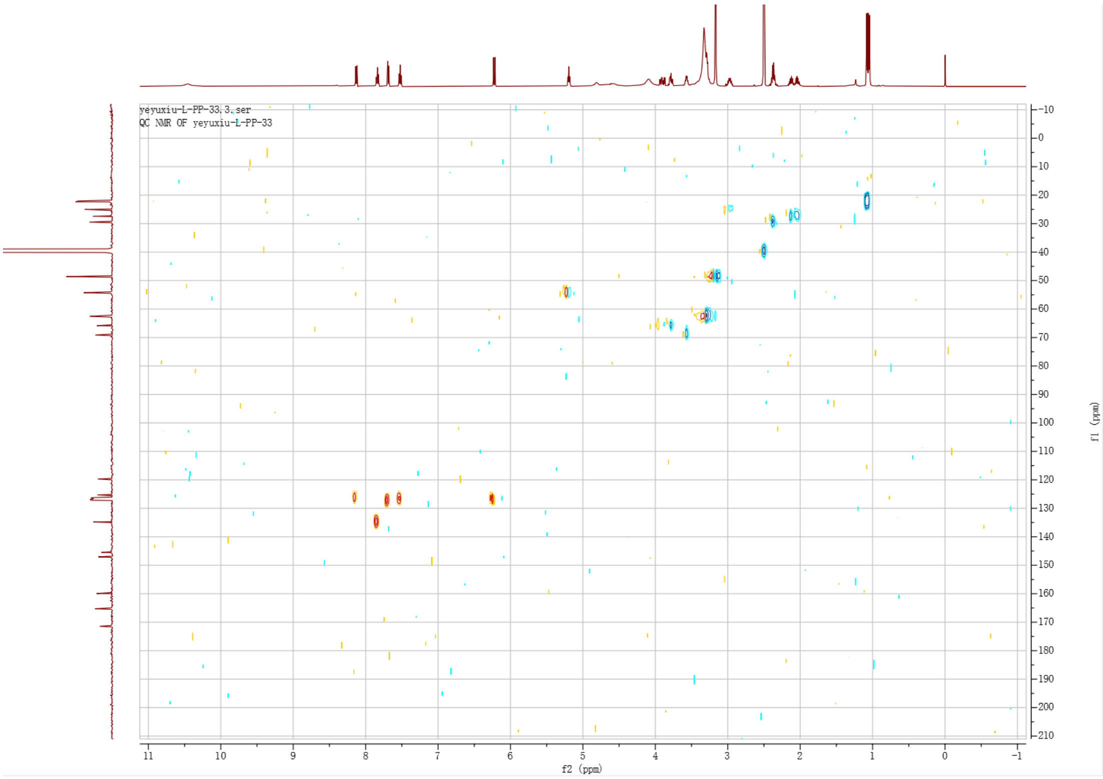

Figure S12. The HSQC spectrum of compound 2 in DMSO.

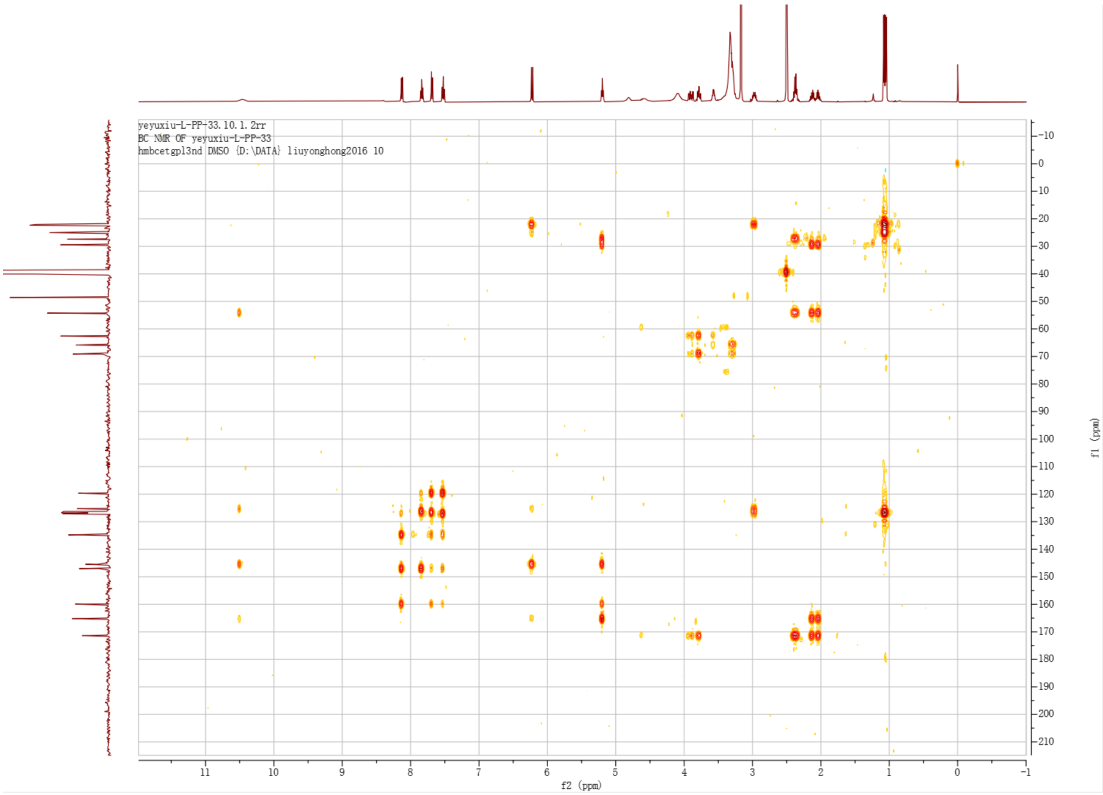

Figure S13. The HMBC spectrum of compound 2 in DMSO.

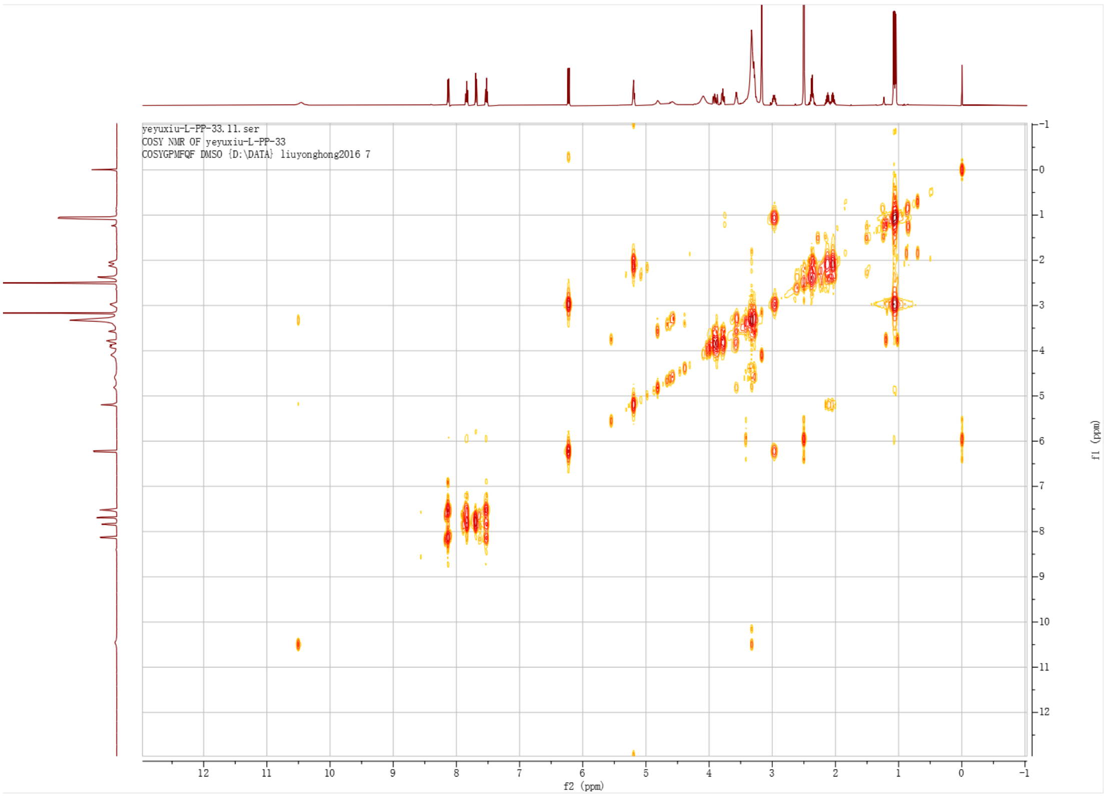

Figure S14. The ¹H-¹H COSY spectrum of compound 2 in DMSO.

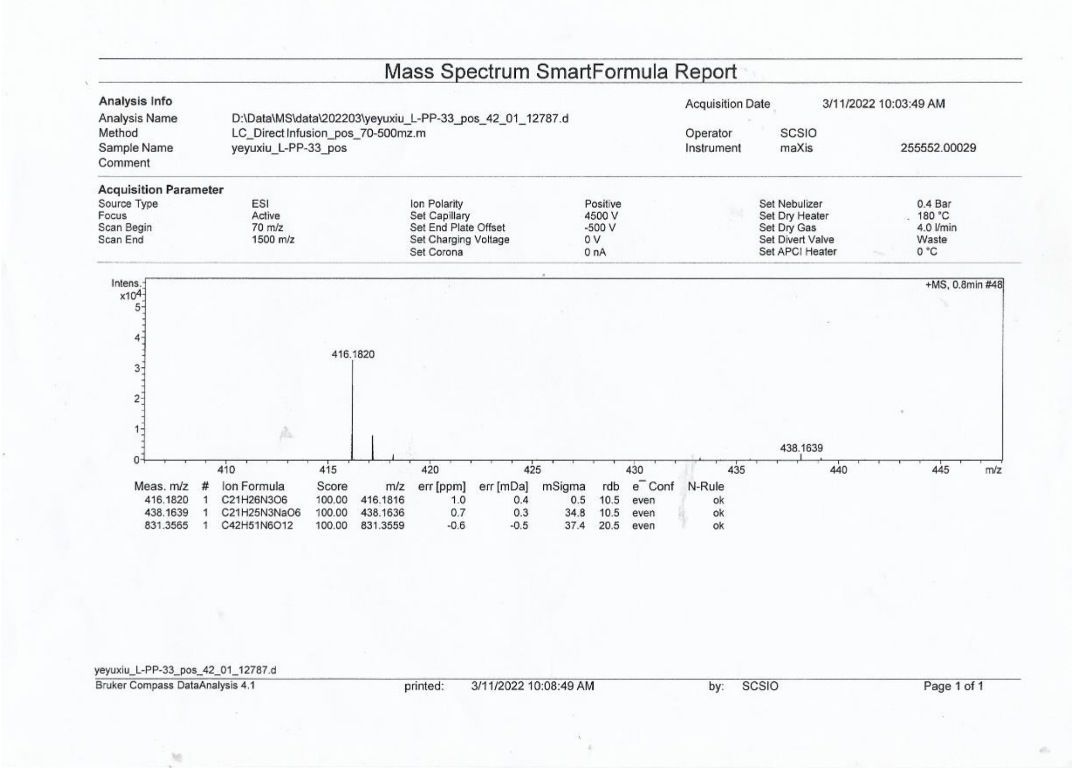

Mass Spectrum SmartFormula Report

Analysis Info

Analysis Name	D:\Data\MS\data\202203\yeyuxiu_L-PP-33_pos_42_01_12787.d	Acquisition Date	3/11/2022 10:03:49 AM
Method	LC_Direct Infusion_pos_70-500mz.m	Operator	SCSIO
Sample Name	yeyuxiu_L-PP-33_pos	Instrument	maXis 255552.00029
Comment			

Acquisition Parameter

Source Type	ESI	Ion Polarity	Positive	Set Nebulizer	0.4 Bar
Focus	Active	Set Capillary	4500 V	Set Dry Heater	180 °C
Scan Begin	70 m/z	Set End Plate Offset	-500 V	Set Dry Gas	4.0 l/min
Scan End	1500 m/z	Set Charging Voltage	0 V	Set Divert Valve	Waste
		Set Corona	0 nA	Set APCI Heater	0 °C

Meas. m/z	#	Ion Formula	Score	m/z	err [ppm]	err [mDa]	mSigma	rdb	e⁻ Conf	N-Rule
416.1820	1	C21H26N3O6	100.00	416.1816	1.0	0.4	0.5	10.5	even	ok
438.1639	1	C21H25N3NaO6	100.00	438.1636	0.7	0.3	34.8	10.5	even	ok
831.3565	1	C42H51N6O12	100.00	831.3559	-0.6	-0.5	37.4	20.5	even	ok

yeyuxiu_L-PP-33_pos_42_01_12787.d
Bruker Compass DataAnalysis 4.1 printed: 3/11/2022 10:08:49 AM by: SCSIO Page 1 of 1

Figure S15. The HRESIMS spectrum of compound 2 in DMSO.

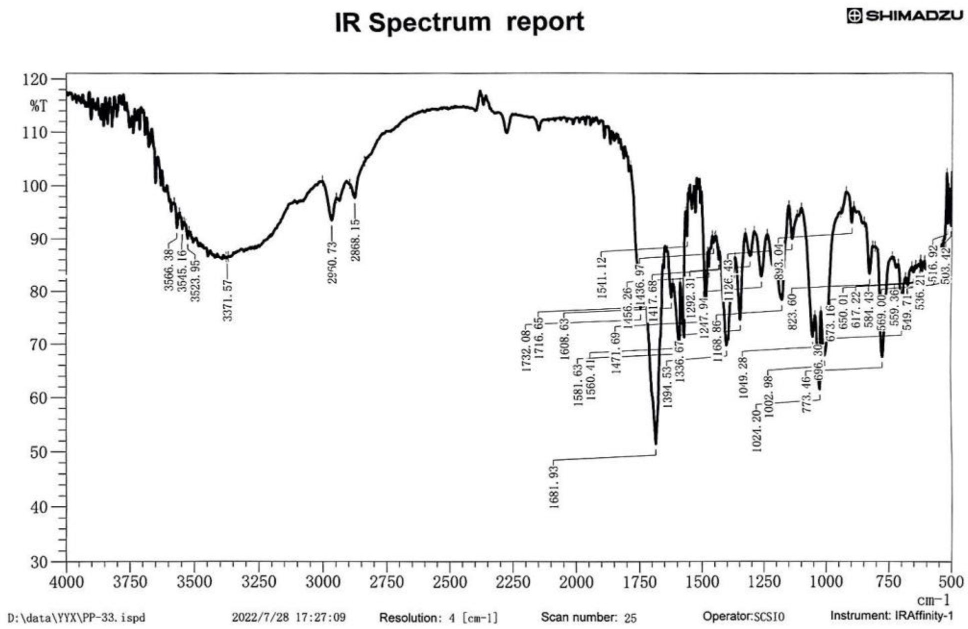

Figure S16. The IR spectrum of compound 2 in DMSO.

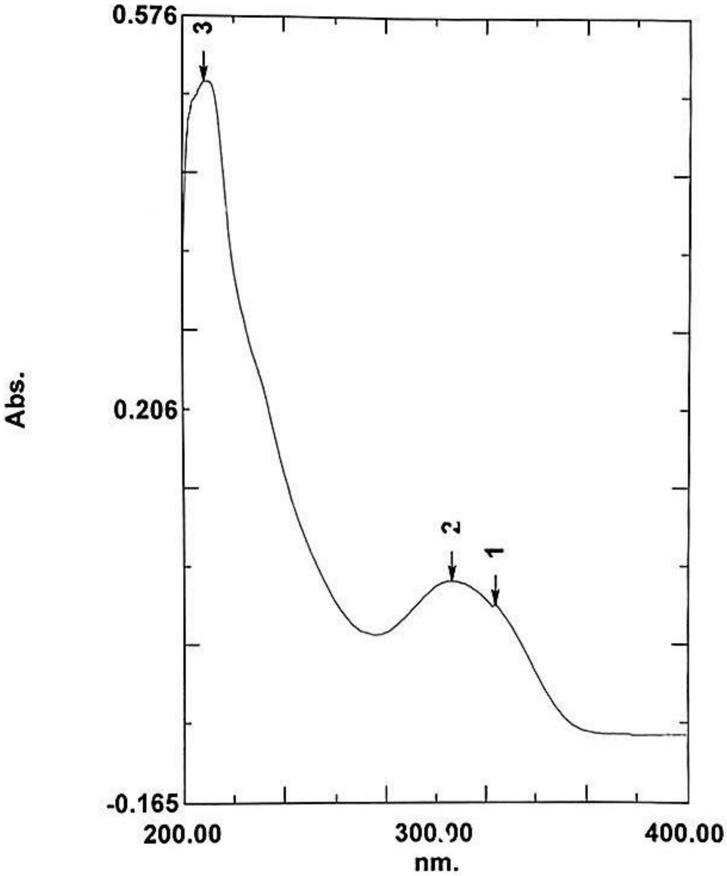

Figure S17. The UV spectrum of compound 2 in DMSO.

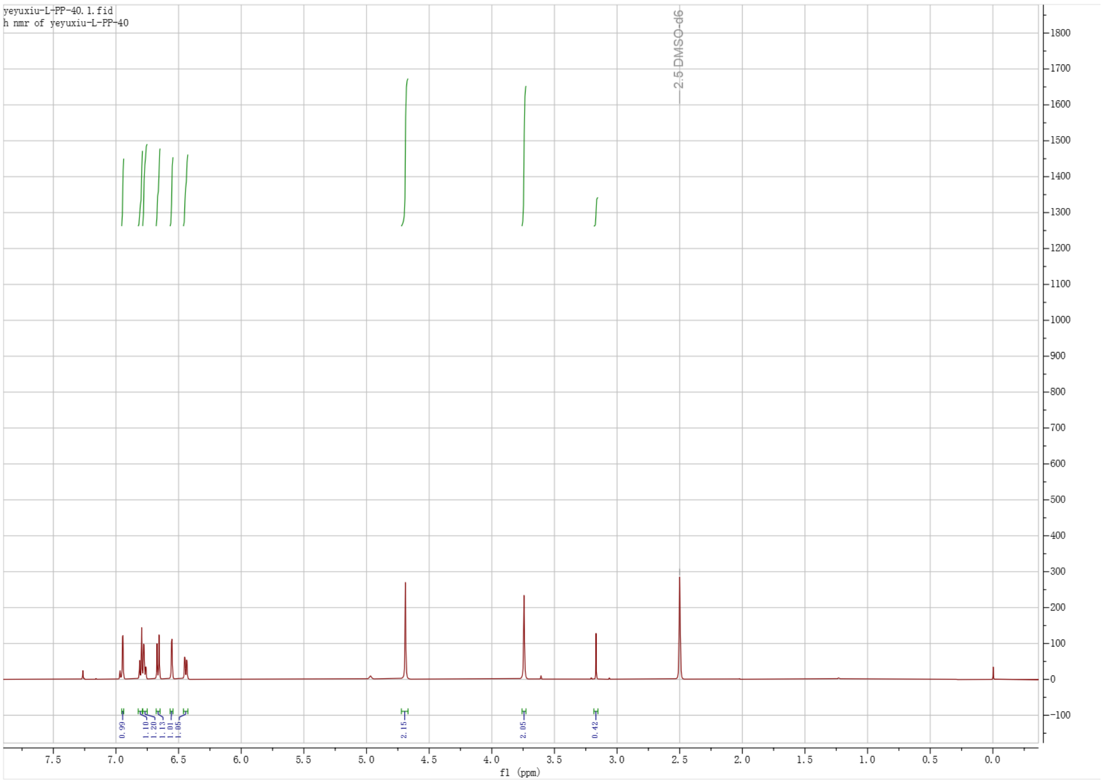

Figure S18. The ^{1}H NMR spectrum of compound 11 in DMSO.

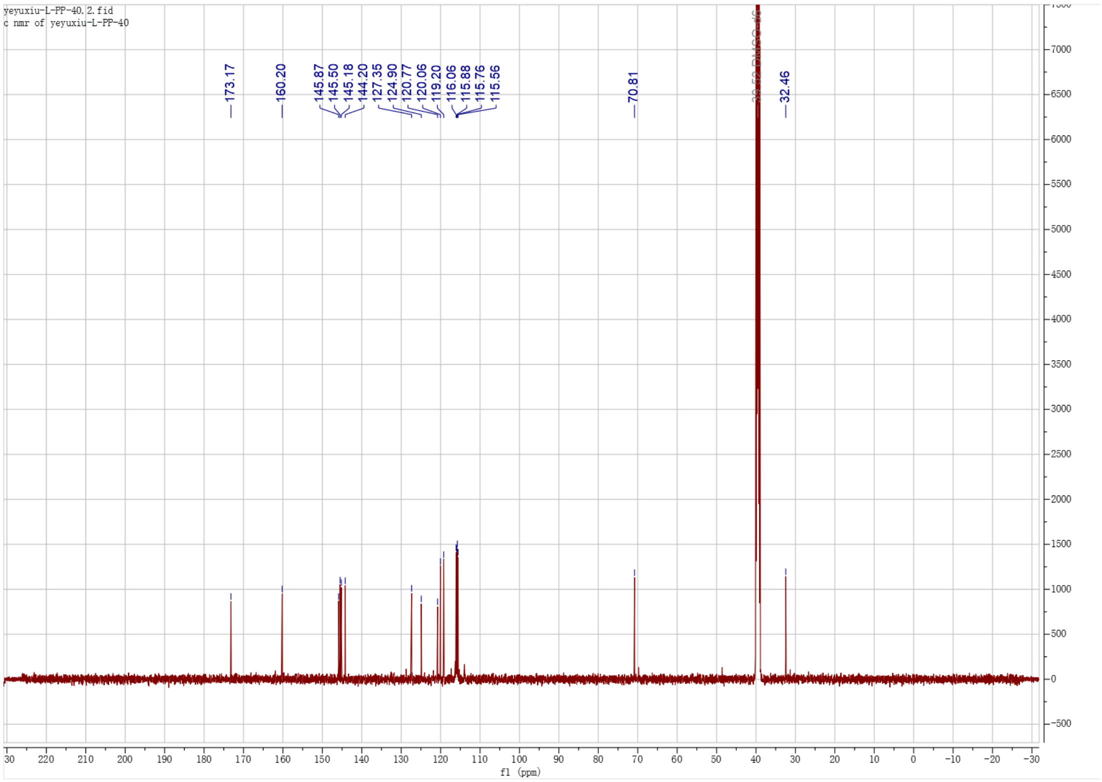

Figure S19. The ^{13}C NMR spectrum of compound 11 in DMSO.

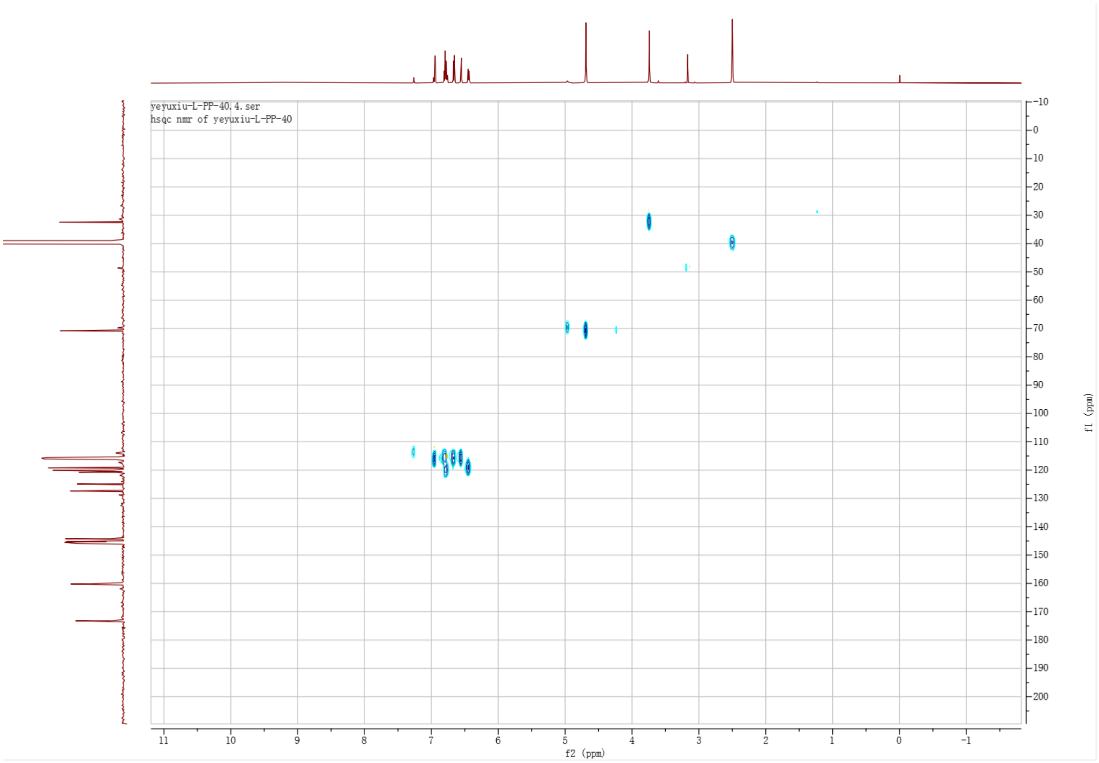

Figure S20. The HSQC spectrum of compound 11 in DMSO.

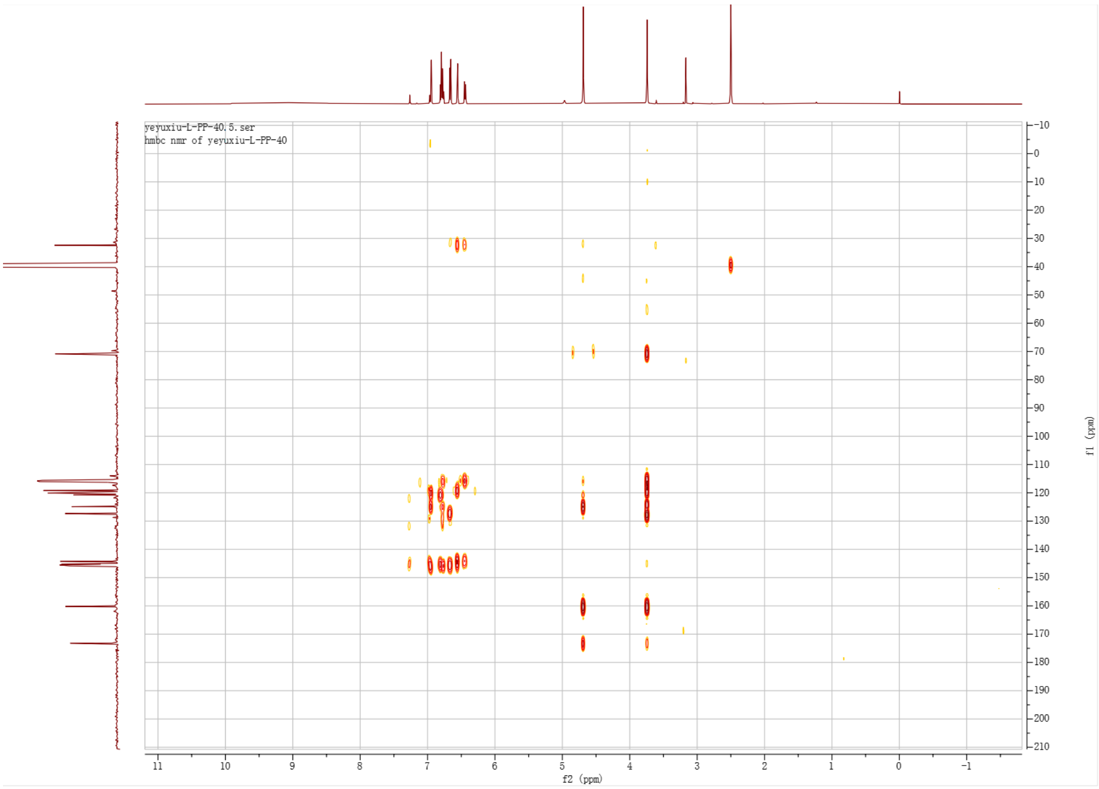

Figure S21. The HMBC spectrum of compound 11 in DMSO.

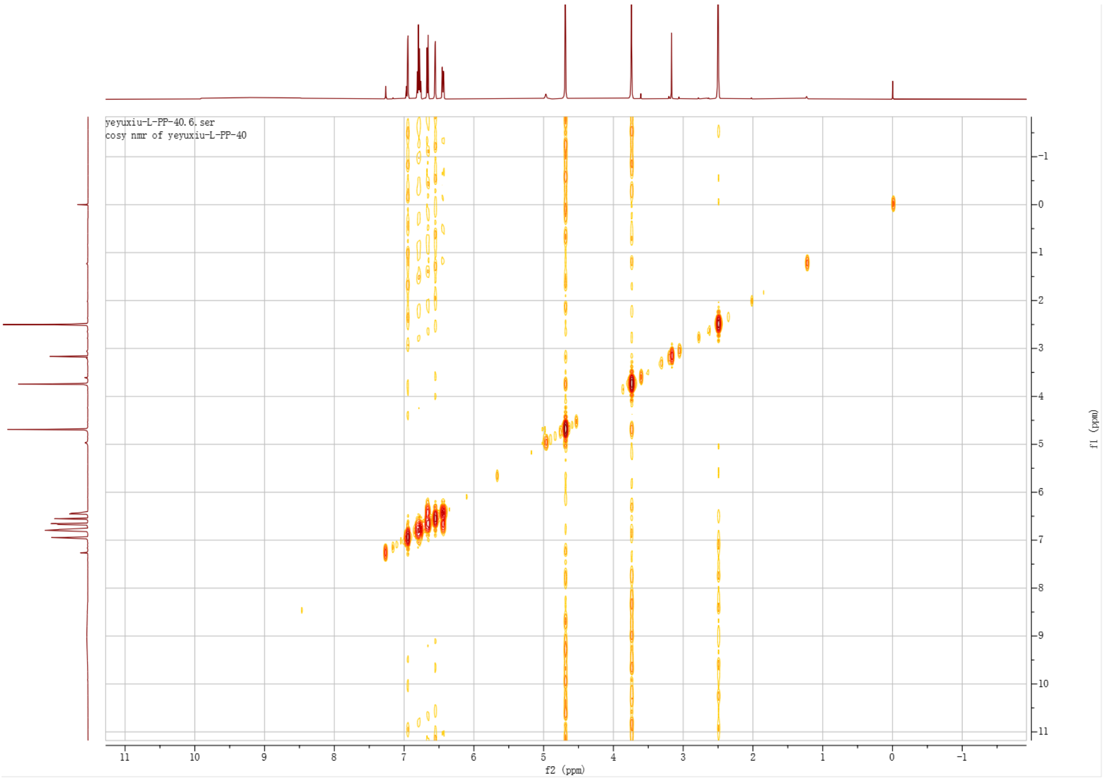

Figure S22. The ^{1}H-^{1}H COSY spectrum of compound 11 in DMSO.

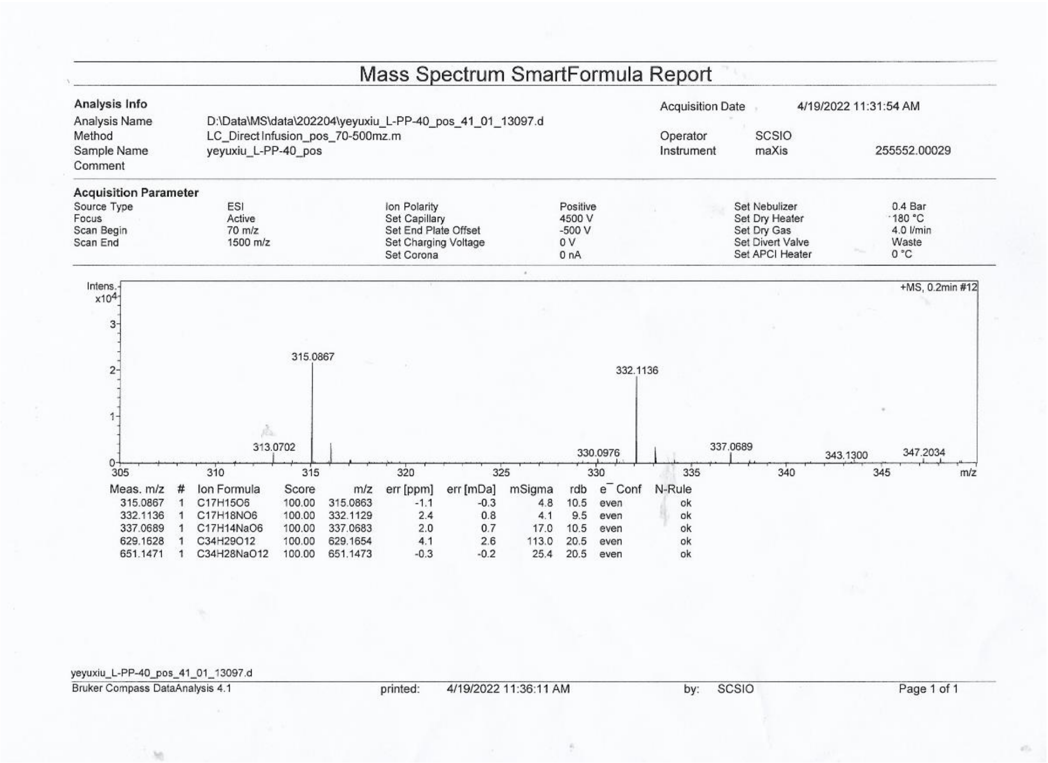

Mass Spectrum SmartFormula Report

Analysis Info

Analysis Name D:\Data\MS\data\202204\yeyuxiu_L-PP-40_pos_41_01_13097.d
Method LC_Direct Infusion_pos_70-500mz.m
Sample Name yeyuxiu_L-PP-40_pos
Comment

Acquisition Date 4/19/2022 11:31:54 AM
Operator SCSIO
Instrument maXis 255552.00029

Acquisition Parameter

Source Type	ESI	Ion Polarity	Positive	Set Nebulizer	0.4 Bar
Focus	Active	Set Capillary	4500 V	Set Dry Heater	180 °C
Scan Begin	70 m/z	Set End Plate Offset	-500 V	Set Dry Gas	4.0 l/min
Scan End	1500 m/z	Set Charging Voltage	0 V	Set Divert Valve	Waste
		Set Corona	0 nA	Set APCI Heater	0 °C

Meas. m/z	#	Ion Formula	Score	m/z	err [ppm]	err [mDa]	mSigma	rdb	e⁻ Conf	N-Rule
315.0867	1	C17H15O6	100.00	315.0863	-1.1	-0.3	4.8	10.5	even	ok
332.1136	1	C17H18NO6	100.00	332.1129	2.4	0.8	4.1	9.5	even	ok
337.0689	1	C17H14NaO6	100.00	337.0683	2.0	0.7	17.0	10.5	even	ok
629.1628	1	C34H29O12	100.00	629.1654	4.1	2.6	113.0	20.5	even	ok
651.1471	1	C34H28NaO12	100.00	651.1473	-0.3	-0.2	25.4	20.5	even	ok

yeyuxiu_L-PP-40_pos_41_01_13097.d
Bruker Compass DataAnalysis 4.1 printed: 4/19/2022 11:36:11 AM by: SCSIO Page 1 of 1

Figure S23. The HRESIMS spectrum of compound 11 in DMSO.

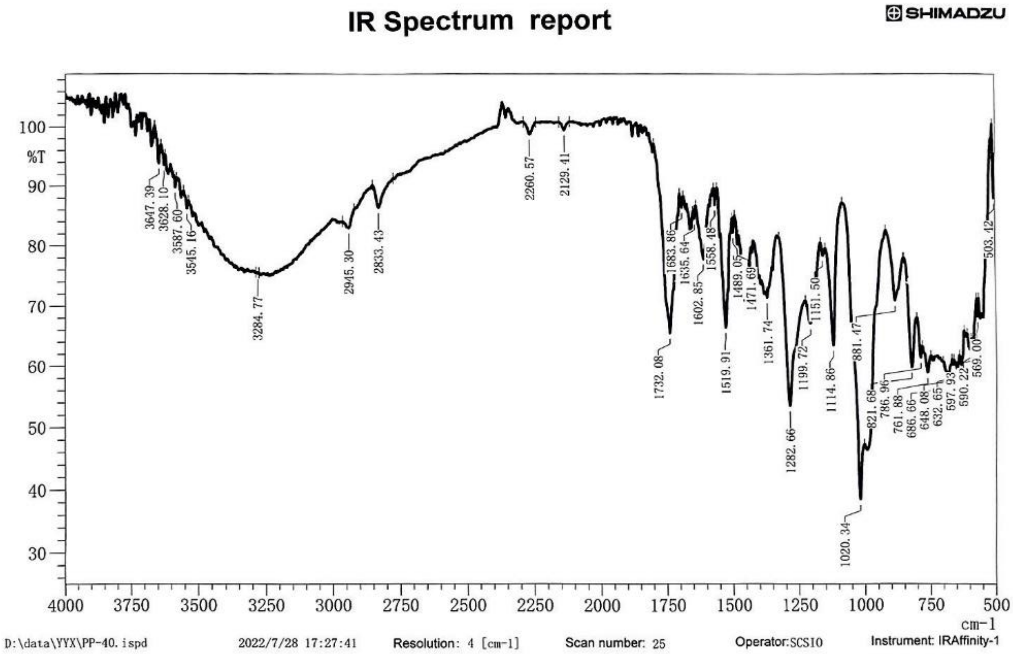

Figure S24. The IR spectrum of compound 11 in DMSO.

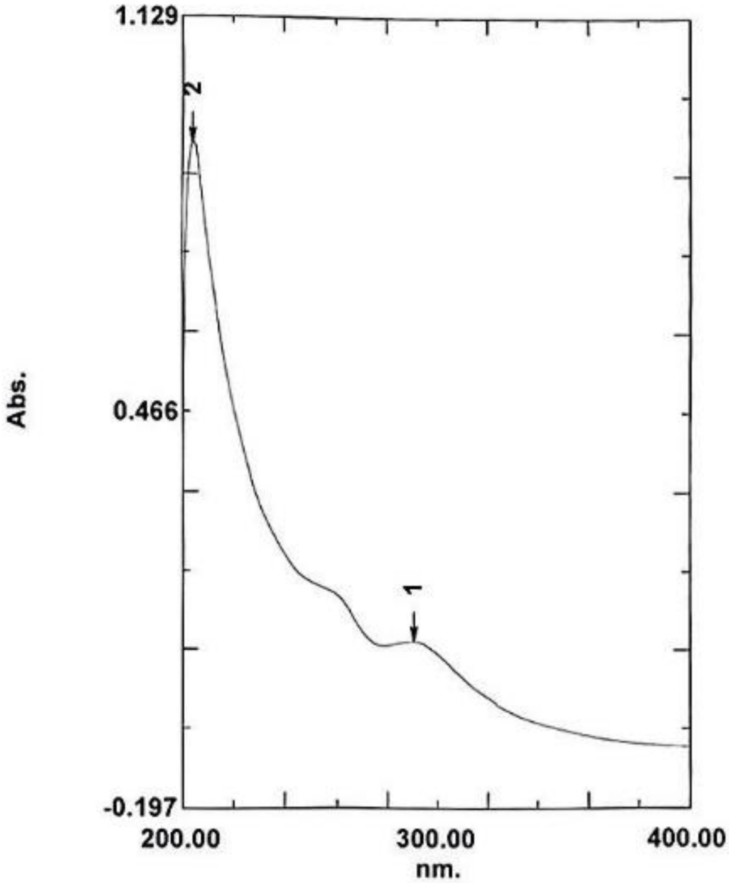

Figure S25. The UV spectrum of compound 11 in DMSO.

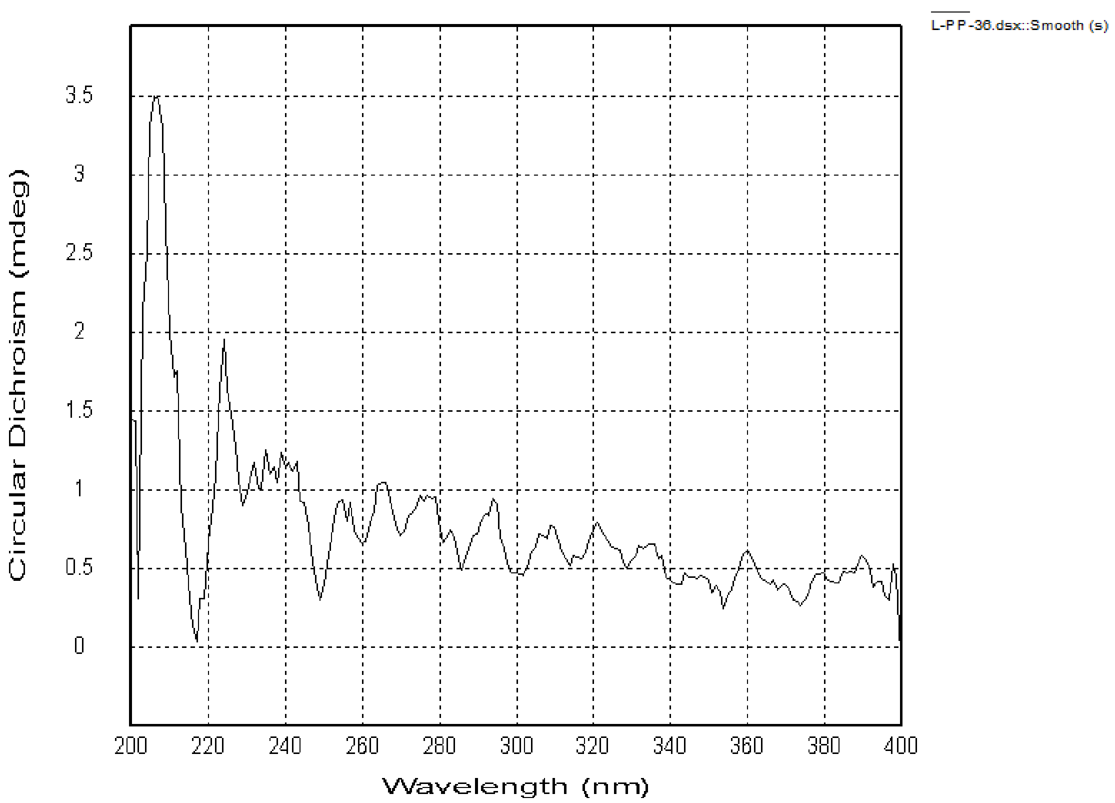

Figure S26. The experimental ECD spectrum of compound 12 in DMSO.

The physicochemical data of the known compounds 3~10 and 12~16

polonimide B (**3**): white oil; $[\alpha]_D^{25}$ -5.42 (c 0.04, MeOH), ^{1}H NMR (500 MHz, DMSO-d_6) δ_H 10.49 (1H, s, H-2), 8.16 (1H, dd, *J* = 8.0, 1.5 Hz, H-10), 7.86 (1H, ddd, *J* = 8.5, 7.1, 1.6 Hz, H-8), 7.65 (1H, d, *J* = 8.1 Hz, H-7), 7.57 (1H, t, *J* = 7.6 Hz, H-9), 7.26 (1H, s, 17-NH_2), 6.71 (1H, s, 17-NH_2), 5.54 (1H, d, *J* = 9.8 Hz, H-18), 5.12 (1H, t, *J* = 6.6 Hz, H-14), 3.76 (1H, d, *J* = 9.8, 6.6 Hz, H-19), 2.18-2.07 (1H, m, H-16), 1.20 (3H, d, *J* = 6.6 Hz, H-21), 1.02 (3H, d, *J* = 6.6 Hz, H-20). ^{13}C NMR (126 MHz, DMSO-d_6) δ_C 172.5 (C-17, C), 165.5 (C-1, C), 159.7 (C-12, C), 146.8 (C-6, C), 144.8 (C-4, C), 134.7 (C-8, CH), 131.0 (C-18, CH), 127.5 (C-7, CH), 127.3 (C-9, CH), 126.3 (C-10, CH), 124.8 (C-3, C), 119.8 (C-11, C), 54.7 (C-14, C), 30.9 (C-16, CH_2), 28.0 (C-15, CH_2), 26.5 (C-19, CH), 23.2 (C-20, CH_3), 22.4 (C-21, CH_3).

Polonimide A (**4**): yellow oil; $[\alpha]_D^{25}$ -33.37 (c 0.1, MeOH), ^{1}H NMR (500 MHz, $CDCl_3$) δ_H 8.26 (1H, dd, *J* = 8.1, 1.5 Hz, H-10), 7.76 (1H, ddd, *J* = 8.5, 7.0, 1.5 Hz, H-8), 7.69 (1H, d, *J* = 7.7 Hz ,H-7), 7.50-7.42 (1H, m, H-9), 6.49 (1H, d, *J* = 10.1 Hz, H-18), 5.51 (1H, t, *J* = 6.8 Hz, H-14), 3.57 (3H, s, 17-OCH_3), 2.83 (1H, q, *J* = 10.0, 8.2 Hz, H-19), 2.54 (2H, dd, *J* = 8.7, 6.7 Hz, H-16),

2.31-2.26 (2H, m, H-15), 1.21 (3H, d, J = 6.5 Hz, H-21), 1.19 (3H, d, J = 6.4 Hz, H-20). ^{13}C NMR (126 MHz, $CDCl_3$) δ_C 172.4 (C-17, C), 166.5 (C-1, C), 160.7 (C-12, C), 147.5 (C-6, C), 144.6 (C-4, C), 134.9 (C-8, CH), 128.0 (C-18, CH), 127.7 (C-9, CH), 127.1 (C-3, C), 127.0 (C-7, CH), 124.7 (C-10, CH), 120.2 (C-11, C), 54.7 (C-14, CH), 51.9 (17-OCH_3, CH_3), 30.1 (C-16, CH_2), 28.5 (C-15, CH_2), 26.3 (C-19, CH), 22.5 (C-21, CH_3), 22.4 (C-20, CH_3).

aurantiomide C (**5**): white powder; $[\alpha]_D^{25}$ -16.6 (c 0.1, MeOH), ^{1}H NMR (500 MHz, $CDCl_3$) δ_H 8.25 (1H, d, J = 8.0 Hz, H-10), 7.78 (2H, d, J = 7.6 Hz, H-7, H-8), 7.49 (1H, t, J = 7.1 Hz, H-9), 6.53 (1H, d, J = 9.9 Hz, H-18), 5.49 (1H, s, H-14), 2.70 (1H, s, H-19), 2.47 (2H, s, H-16), 2.24 (2H, s, H-15), 1.21 (6H, d, J = 6.2 Hz, H-20, H-21). ^{13}C NMR (126 MHz, $CDCl_3$) δ_C 174.2 (C-17, C), 166.6 (C-1, C), 160.9 (C-12, C), 147.5 (C-6, C), 144.8 (C-4, C), 134.9 (C-9, C), 128.4 (C-18, CH), 128.4 (C-7, CH), 127.6 (C-9, CH), 127.0 (C-10, CH), 126.9 (C-3, C), 120.0 (C-11, C), 54.8 (C-14, CH), 31.8 (C-16, CH_2), 29.0 (C-15, CH_2), 26.2 (C-19, CH), 22.5 (C-20, CH_3), 22.4 (C-21, CH_3).

fructigenine A (**6**): white powder; $[\alpha]_D^{25}$ -31.4 (c 0.1, MeOH), ^{1}H NMR (500 MHz, DMSO-d_6) δ_H 8.20 (1H, s, 2-NH), 7.84 (1H, d, J = 8.1 Hz, H-7), 7.38 (1H, dd, J = 7.7, 1.3 Hz, H-8), 7.31 – 7.22 (6H, m, H-10, 14, 15, 16, 17, 18), 7.13 (1H, d, J = 7.5, 1.1 Hz, H-9), 5.92 (1H, s, H-5a), 5.64 (1H, dd, J = 17.3, 10.8 Hz, H-20), 5.06-5.01 (2H, m, H-21), 4.44 (1H, t, J = 4.6 Hz, H-13), 3.60-3.53 (1H, m, H-11), 3.12 (1H, dd, J = 14.0, 4.3 Hz, Ha-12), 3.01 (1H, dd, J = 14.1, 5.1 Hz, Hb-12), 2.55 (3H, s, H_3-23), 2.32 (1H, dd, J = 12.3, 5.3 Hz, Ha-11), 1.61 (1H, t, J = 12.0 Hz, Hb-11), 0.94 (3H, s, H_3-19a), 0.79 (3H, s, H_3-19b). ^{13}C NMR (126 MHz, DMSO-d_6) δ_C 169.5 (C-22, C), 166.6 (C-4, C), 165.2 (C-1, C), 143.4 (C-7a, C), 143.2 (C-20, C), 136.6 (C-13, C), 132.5 (C-10a, C), 130.0 (C-15, 17, CH), 128.4 (C-8, CH), 128.1 (C-14, 18, CH), 126.5 (C-16, CH), 124.9 (C-10, CH), 124.1 (C-7, CH), 117.7 (C-9, CH), 114.1 (C-21, CH_2), 78.6 (C-5a, CH), 60.5 (C-10b, CH), 58.5 (C-11b, CH), 55.5 (C-3, CH), 40.1 (C-19, C),36.3 (C-11, CH_2), 36.0 (C-12, CH_2), 23.7 (C-19b, CH_3), 22.9 (C-19a, CH_3), 22.1 (C-23, CH_3).

3-O-methylviridicatin (**7**): white powder; 1H NMR (500 MHz, Chloroform-d) δ_H 7.53 (2H, t, J = 7.2 Hz, H-2',6'), 7.48 (1H, d, J = 7.2 Hz, H-4'), 7.43 (2H, m, H-7,8), 7.37 (2H, d, J = 7.2 Hz, H-3',5'), 7.23 (1H, d, J = 8.0 Hz, H- 5), 7.11 (1H, m, H-6), 3.83 (3H, s, 3-OCH_3). ^{13}C NMR (126 MHz, DMSO) δ_C 158.5 (C-2, C), 145.1 (C-3, C), 137.4 (C-4, CH), 135.8 (C-8a, C), 133.5 (C-1', C),

129.1 (C-2’, CH), 129.1 (C-6’, CH) 128.6 (C-7, CH), 128.4 (C-3’, CH), 128.4 (C-5’, CH),128.0 (C-4’, CH), 125.7 (C-5, CH), 122.0 (C-6, CH), 119.8 (C-4a, C), 115.1 (C-8, C), 59.4 (3-OCH_3, CH_3).

viridicatol (**8**): yellow powder; ^{1}H NMR (500 MHz, DMSO-d_6) δ_H 12.19 (1H, s, 2-OH), 9.52 (1H, s, 3-OH), 9.14 (1H, s, 3’-OH), 7.35-7.27 (3H, m, H-8,5,2’), 7.13-7.04 (2H, m, H-6, 7), 6.85-6.79 (1H, m, H-4), 6.74-6.69 (2H, m, H-5’,6’). ^{13}C NMR (126 MHz, DMSO) δ_C 158.3 (C-2, C), 157.3 (C-3’, C), 142.3 (C-3, C), 134.9 (C-4, C), 133.1 (C-9, C), 129.4 (C-2’, CH), 126.4 (C-5, CH), 124.4 (C-7, CH), 124.0 (C-1’, C), 122.1 (C-6, CH), 120.9 (C-10, C), 120.4 (C-5’, CH), 116.7 (C-6’, CH), 115.2 (C-8, CH), 114.6 (C-4’, CH).

arctosin (**9**): yellow oil; ^{1}H NMR (500 MHz, DMSO-d_6) δ_H 7.53 (1H, ddd, *J* = 8.4, 7.5, 1.7 Hz, H-8), 7.15 (1H, dd, *J* = 8.4, 1.1 Hz,H-9), 7.09 (1H, td, *J* = 7.5, 1.1 Hz, H-7), 7.02-6.98 (1H, m, H-6), 6.96 (1H, d, *J* = 7.9 Hz, H-15), 6.69-6.65 (1H, m, H-14), 6.11 (1H, t, *J* = 2.0 Hz, H-12), 6.02-5.98 (1H, m, H-16), 4.23 (1H, s, H-10), 3.06 (3H, s, 4-CH_3). ^{13}C NMR (126 MHz, DMSO- d_6) δ_C 166.0 (C-2, C), 165.4 (C-5, C), 157.0 (C-13, C), 135.1 (C-9a, C), 132.4 (C-8, CH), 132.3 (C-11, C), 130.5 (C-6, CH), 128.9 (C-15, CH), 126.5 (C-5a, C), 124.2 (C-7, CH), 121.1 (C-9, CH), 116.9 (C-16, CH), 115.7 (C-14, CH), 112.8 (C-12, CH), 70.1 (C-3, C), 63.7 (C-10, C), 30.9 (4-CH_3, CH_3).

cyclopenin (**10**): yellow oil; $[\alpha]_D^{25}$ -60.1 (c 0.05, MeOH), ^{1}H NMR (500 MHz, DMSO-d_6) δ_H 10.84 (1H, s, -NH), 7.54 (1H, ddd, *J* = 8.4, 7.7, 1.6 Hz, H-8), 7.32-7.28 (1H, m, H-14), 7.22 (2H, t, *J* = 7.7 Hz, H-13, 15), 7.16 (1H, dd, *J* = 8.4, 1.1 Hz, H-9), 7.09 (1H, td, *J* = 7.7, 1.1 Hz, H-7), 6.92 (1H, dd, *J* = 7.7, 1.6 Hz, H-6), 6.66-6.61 (2H, m, H-12, 16), 4.36 (1H, s, H-10), 3.32 (3H, s, 4-CH_3). ^{13}C NMR (126 MHz, DMSO-d_6) δ_C 165.9 (C-2, C), 165.3 (C-5, C), 135.2 (C-9a, C), 132.4 (C-8, CH), 131.0 (C-11, C), 130.5 (C-9, CH), 128.7 (C-14, CH), 127.9 (C-13, CH), 127.9 (C-15, CH), 126.4 (C-5a, C), 126.1 (C-12, CH), 126.1 (C-16, CH), 124.2 (C-7, CH), 121.1 (C-6, CH), 70.1 (C-3, C), 63.7 (C-10, CH), 30.8 (4-CH_3, CH_3).

8-Hydroxyhelvafuranone (**12**): green oil; $[\alpha]_D^{25}$ + 3.07 (c 0.05, MeOH), ^{1}H NMR (500 MHz, DMSO-d_6) δ_H 9.33 (1H, s, 8-OH), 9.22 (1H, s, 9-OH), 9.15 (1H, s, 16-OH), 7.80 (1H, d, *J* = 8.3 Hz,5-OH), 6.99 (2H, d, *J* = 8.1 Hz, H-14, 18), 6.93 (1H, d, *J* = 2.0 Hz, H-7), 6.80 (1H, d, *J* = 8.1 Hz, H-10), 6.76 (1H, dd, *J* = 8.2, 2.0 Hz, H-11), 6.70 (2H, d, *J* = 8.2 Hz, H-15, 17), 5.78 (1H, d, *J* = 7.7 Hz, H-5), 3.85 (1H, d, *J* = 15.1 Hz, H-12), 3.58 (1H, d, *J* = 15.1 Hz, H-12). ^{13}C NMR (126 MHz, DMSO-d_6) δ_C 170.6 (C-2, C), 157.7 (C-4, C), 156.1 (C-16, C), 146.1 (C-9, C), 145.1 (C-8,

C), 129.6 (C-14, 18, CH), 127.8 (C-6, C), 126.5 (C-13, C), 120.3 (C-3, C), 120.2 (C-11, CH), 116.1 (C-7, CH), 115.6 (C-10, CH), 115.5 (C-15, 17,CH), 96.5 (C-5, CH), 31.0 (C-12, CH_2).

verrucosidinol acetate (**13**): white oil; $[\alpha]_D^{25}$ +9.7 (c 0.03, MeOH), ^{1}H NMR (500 MHz, DMSO-d_6) δ_H 5.75 (1H, d, J = 3.2 Hz, H-9), 5.69 (1H, s, H-7), 5.38 (1H, s, H-11), 3.96 (1H, d, J = 6.9, 3.2 Hz, H-15), 3.74 (3H, t, J = 4.2 Hz, H-24), 3.55 (1H, d, J = 3.2 Hz, H-13), 2.17 (3H, t, J = 4.2 Hz, H-25), 2.00 (3H, d, J = 3.2 Hz, H-17), 1.89 (3H, d, J = 3.4 Hz, H-16), 1.78 (3H, d, J = 3.0 Hz, H-20), 1.73 (3H, d, J = 3.0 Hz, H-19), 1.44 (3H, d, J = 3.2 Hz, H-18), 1.38 (3H, d, J = 3.2 Hz, H-22), 1.23 (3H, d, J = 3.1 Hz, H-21), 1.05 (3H, dd, J = 7.2, 3.1 Hz, H-23). ^{13}C NMR (126 MHz, DMSO) δ_C 169.2 (C-26, C), 168.4 (C-3, C), 163.6 (C-1, C), 158.4 (C-5, C), 133.8 (C-9, CH), 133.7 (C-10, C), 132.8 (C-11, CH), 130.9 (C-8, C), 111.6 (C-4, C), 109.3 (C-2, C), 81.6 (C-7, CH), 79.3 (C-12, C), 77.5 (C-6, C), 75.8 (C-15, CH), 66.9 (C-13, CH), 66.3 (C-14, C), 60.2 (C-24, C), 23.3 (C-18, CH_3), 21.9 (C-21, CH_3), 20.8 (C-25, CH_3), 18.6 (C-23, CH_3), 18.1 (C-22, CH_3), 15.1 (C-19, CH_3), 13.5 (C-22, CH_3), 9.9 (C-16, CH_3), 9.8 (C-17, CH_3).

deoxyverrucosidin (**14**): yellow oil; $[\alpha]_D^{25}$ -0.1 (c 0.1, MeOH), ^{1}H NMR (500 MHz, $CDCl_3$) δ_H 6.15 (1H, s, H-7), 5.79 (1H, s, H-9), 5.36 (1H, s, H-11), 4.10 (1H, q, J = 6.8 Hz, H-15), 3.81 (3H, s, H-18), 3.40(1H, s, H-13), 2.07(3H, s, H-17), 2.00 (3H, d, J = 1.5 Hz, H-21), 1.86 (3H, d, J = 1.5 Hz, H-22), 1.82 (3H, s, H-19), 1.66 (3H, d, J = 1.4 Hz, H-20), 1.15 (3H, d, J = 6.8 Hz, H-16).^{13}C NMR (126 MHz, $CDCl_3$) δ_C 168.2 (C-3, C), 166.1 (C-1, C), 156.9 (C-5, C), 137.9 (C-7, CH), 136.9 (C-9, CH), 135.1 (C-8, C), 133.6 (C-11, CH), 132.6 (C-10, C), 126.6 (C-6, C), 110.2 (C-2, C-4, 2C), 80.2 (C-12, C), 76.5 (C-15, CH), 67.6 (C-13, CH), 67.4 (C-14, C), 60.4 (C-18, CH_3), 23.4 (C-23, CH_3), 22.0 (C-16, CH_3), 19.0 (C-21, CH_3), 18.6 (C-22, CH_3), 16.4 (C-20, CH_3), 13.9 (C-24, CH_3), 10.9 (C-19, CH_3), 10.5 (C-17, CH_3).

nordeoxyverrucosidin (**15**): yellow oil; $[\alpha]_D^{25}$ +2.7 (c 0.1, MeOH), ^{1}H NMR (500 MHz, $CDCl_3$) δ 6.07 (1H, s, H-7), 5.86 (1H, s, H-9), 5.51 (1H, d, J = 1.5 Hz, H-11), 5.48 (1H, s, H-2),4.14 (1H, q, J = 6.8 Hz, H-15), 3.83 (3H, s, 3-OCH_3), 3.44 (1H, s, H-13), 2.07 (3H, d, J = 1.5 Hz, 6-CH_3), 1.96 (9H, dd, J = 3.6, 2.1 Hz, 4-CH_3, 8-CH_3, 10-CH_3), 1.48 (3H, s, 14-CH_3), 1.43(3H, s, 12-CH_3) 1.21 (3H, d, J = 6.8 Hz, 15-CH_3). ^{13}C NMR (126 MHz, $CDCl_3$) δ_C 171.5 (C-3, C), 164.4, (C-1, C) 161.5 (C-5, C), 139.4 (C-7, CH), 136.5 (C-9, CH), 135.1 (C-8, C), 133.6 (C-11, CH), 132.1 (C-10, C), 127.5 (C-6, C), 106.8 (C-4, C), 88.3 (C-2, CH), 80.3 (C-12, C), 76.9 (C-15, CH),67.6 (C-13, CH),

67.6 (C-14, C), 56.3 (3-OCH_3, CH_3), 22.0 (12-CH_3, CH_3), 19.0 (15-CH_3, CH_3), 18.7 (10-CH_3, CH_3), 18.6 (8-CH_3, CH_3), 16.7 (6-CH_3, CH_3), 14.0 (14-CH_3, CH_3), 11.2 (4-CH_3, CH_3).

aspterric acid methyl ester (**16**): yellow oil; $[\alpha]_D^{25}$ -9.23 (c 0.1, MeOH) ^{1}H NMR (500 MHz, Chloroform-d) δ_H 4.17 (1H, d, J = 8.4 Hz, H-2), 3.79 (3H, s, H-16), 3.37 (1H, d, *J* = 8.2 Hz, H-13a), 2.46-2.40 (1H, m, H-13b), 2.37-2.32 (2H, m, H-4), 2.28 (1H, d, *J* = 8.3 Hz, H-6), 2.18 (1H, dd, *J* = 13.2, 8.4 Hz, H-1a), 2.04 (1H, d, *J* = 13.3 Hz, 8.4 Hz, H-1b), 2.01 (1H, d, *J* = 5.5 Hz, H-9a), 1.84-1.76 (1H, m, H-5a), 1.74 (3H, s, H-14), 1.70 (1H, d, *J* = 8.3 Hz, H-5b), 1.60 (3H, s, H-15), 1.47 (1H, d, *J* = 5.5 Hz, H-9b), 1.18 (1H, dt, *J* = 14.0, 6.6 Hz, H-8a), 1.03 (1H,d, *J* = 14.0, 6.6 Hz, H-8b). ^{13}C NMR (126 MHz, Chloroform-d) δ_C 176.2 (C-12, C), 135.5 (C-10, C), 124.6 (C-11, C), 84.9 (C-2, CH), 78.6 (C-3, C), 76.2 (C-13, CH_2), 55.7 (C-6, CH), 53.1 (C-7, C), 52.6 (C-16, CH_3), 36.3 (C-1, CH_2), 34.4 (C-4, CH_2), 33.4 (C-8, CH_2), 32.4 (C-9, CH_2), 23.7 (C-5, CH_2), 23.5 (C-15, CH_3), 21.0 (C-14, CH_3).
